# Peptide Bond Formation between Aminoacyl-Minihelices by a Scaffold Derived from the Peptidyl Transferase Center

**DOI:** 10.3390/life12040573

**Published:** 2022-04-12

**Authors:** Mai Kawabata, Kentaro Kawashima, Hiromi Mutsuro-Aoki, Tadashi Ando, Takuya Umehara, Koji Tamura

**Affiliations:** 1 Department of Biological Science and Technology, Tokyo University of Science, 6-3-1 Niijuku, Katsushika-ku, Tokyo 125-8585, Japan; bata2.u.u.snail@gmail.com (M.K.); 8315040@alumni.tus.ac.jp (K.K.); mutsuro@rs.tus.ac.jp (H.M.-A.); t.ummechi@gmail.com (T.U.); 2 Department of Applied Electronics, Tokyo University of Science, 6-3-1 Niijuku, Katsushika-ku, Tokyo 125-8585, Japan; tando@rs.tus.ac.jp; 3 Research Institute for Science and Technology, Tokyo University of Science, 2641 Yamazaki, Noda, Chiba 278-8510, Japan

**Keywords:** tRNA, minihelix, peptide bond formation, ribosomal symmetrical region, proto-ribosome

## Abstract

The peptidyl transferase center (PTC) in the ribosome is composed of two symmetrically arranged tRNA-like units that contribute to peptide bond formation. We prepared units of the PTC components with putative tRNA-like structure and attempted to obtain peptide bond formation between aminoacyl-minihelices (primordial tRNAs, the structures composed of a coaxial stack of the acceptor stem on the T-stem of tRNA). One of the components of the PTC, P1c2^UGGU^ (74-mer), formed a dimer and a peptide bond was formed between two aminoacyl-minihelices tethered by the dimeric P1c2^UGGU^. Peptide synthesis depended on both the existence of the dimeric P1c2^UGGU^ and the sequence complementarity between the ACCA-3′ sequence of the minihelix. Thus, the tRNA-like structures derived from the PTC could have originated as a scaffold of aminoacyl-minihelices for peptide bond formation through an interaction of the CCA sequence of minihelices. Moreover, with the same origin, some would have evolved to constitute the present PTC of the ribosome, and others to function as present tRNAs.

## 1. Introduction

**Figure 1:**
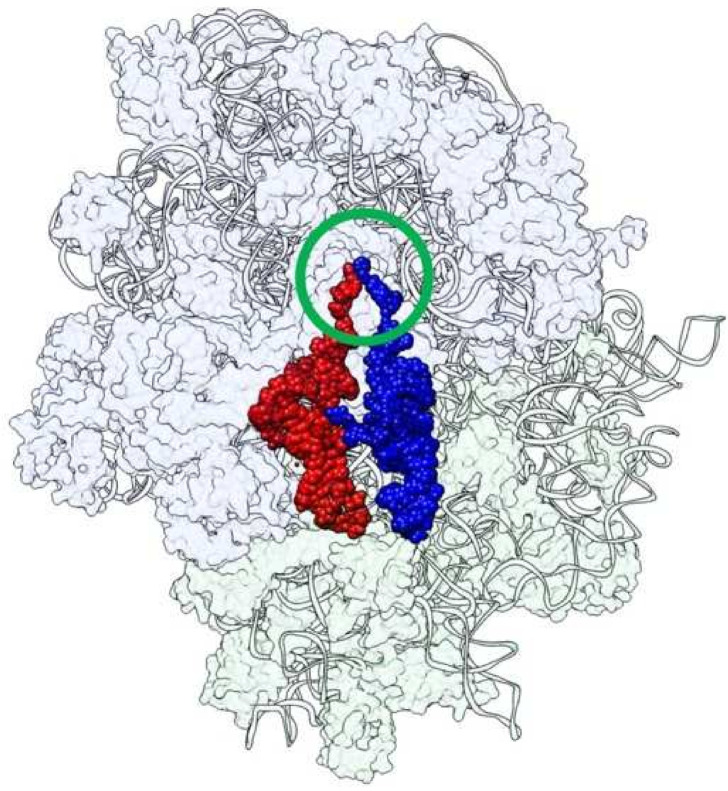
The structure of the *Thermus thermophilus* 70S ribosome in complex with P-site tRNA (red) and A-site tRNA (blue) (PDB ID: 4V5D). The two CCA termini (circled in green) approach in close proximity to perform peptide bond formation, just like a “Goromaru pose” [10].

The ribosome is composed of a large and a small subunit. In the large subunit, peptide bond formation occurs at the peptidyl transferase center (PTC), and in the small subunit, the anticodon of tRNA corresponding to the codon information of mRNA is decoded [1,2]. Peptide bond formation occurs in a non-amino acid-specific manner, which is distinct from the codon–anticodon pairing between mRNA and tRNA. Therefore, it is speculated that the large subunit appeared evolutionarily before the small subunit [3]. The PTC is composed of only an RNA molecule, and the closest proteins are detected approximately 18 Å away [4,5]. This suggests that the ribosome is a ribozyme [6,7]. The CCA-3′ termini of two tRNA molecules specifically interact with domain V of the bacterial/archaeal 23S rRNA, and the nucleophilic attack of the α-amino group of aminoacyl-tRNA on the carbonyl carbon of peptidyl-tRNA leads to the formation of a peptide bond [5,8]. To ensure the reactions through an entropic effect, the two CCA termini must be positioned in close proximity on the ribosome. Thus, it is important to identify the entity that could have primarily been a template that scaffolds the necessary proximity of two tRNAs in considering the continuity of biological evolution. Based on the structure of the *Thermus thermophilus* 70S ribosome in complex with P- and A-site tRNAs (PDB ID: 4V5D) (Figure 1) [9], an angle between the two single-stranded CCAs is about 40°, which resembles the positioning of the index fingers of a rugby player Goromaru’s routine (“Goromaru pose”) in the moments before a kick attempt [10].

**Figure 2:**
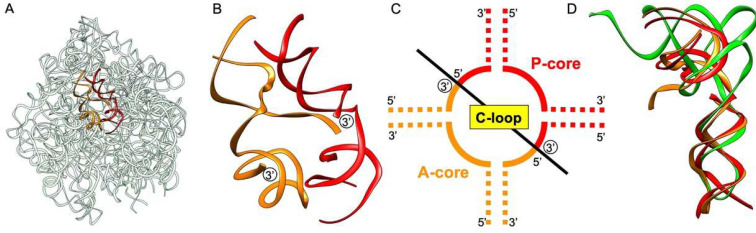
(**A**) The structure of 23S ribosomal RNA of *Deinococcus radiodurans* large subunit (PDB ID: 1NJP). The region of the central loop (C-loop) of domain V composed of the core of P-site (P-core unit) is in red and that composed of the core of A-site (A-core unit) is in orange. (**B**) Close-up image of the P-core unit and A-core unit. The 3′-ends of both units are labeled with circled 3′. (**C**) Schematic representation of symmetrical regions of the C-loop. The 3′-ends of both units are labeled with circled 3′. (**D**) P-core unit (red) and A-core unit (orange) shown in (**A**) overlap well with tRNA (green; PDB ID: 1EHZ). Fitting was performed using RNA-align (https://zhanggroup.org/RNA-align/) (accessed on 22 March 2022).

The 23S rRNA is composed of about 3000 nucleotides, and it was considered that the small primordial ribosome containing the PTC appeared first, which may have helped the transition from the RNA world to modern systems in the course of evolution [3,11]. The crystal structures of both bacterial and archaeal large ribosomal subunits clearly indicate that two L-shaped RNA units, called P1 and A1, similar in size to tRNA, form a symmetrical pocket in the central loop (C-loop) of domain V (Figure 2) [3,11,12,13,14]. P1 is derived from the region of the C-loop composed of the P-site, and A1 is derived from the region of the C-loop composed of the A-site. Of these, P1 of the large subunit of *Deinococcus radiodurans* exhibits self-dimerization in the presence of Mg^2+^, suggesting that it can form a similar symmetrical three-dimensional structure as seen in the ribosome (Figure 2) [15]. Furthermore, P1c, a mutated version of P1, has been reported to display a higher self-dimerization ability [15]. It has also been shown very recently that some combinations of small RNA pocket-like segments derived from P1 and A1 regions are capable of mediating peptide bond formation [16] using the substrates CCA-phenylalanine-caproic acid-biotin (CCA-pcb) and C-puromycin (C-Pmn) [17].

In this study, we attempted to obtain peptide bond formation between aminoacyl-minihelices using modified P1c constructs, expecting to render the RNAs with the same “function” as in the ribosome. Minihelix is composed of a coaxial stack of the acceptor stem on the T-stem of tRNA and is considered to be a primordial tRNA in primitive protein biosynthesis [3,18,19]. The putative primordial PTC formed by dimerized RNAs may have played the role of a scaffold that caused two aminoacyl-tRNAs to be adjacent to each other, facilitating peptide bond formation. We investigated whether aminoacyl-minihelices and modified P1c constructs interact with each other to produce a dipeptide.

## 2. Materials and Methods

### 2.1. In Vitro Transcription

PCR amplified DNAs carrying the T7 promoter and the aimed sequences were used for RNA transcription. The primers and templates were synthesized by Eurofins Genomics K.K. (Tokyo, Japan). RNA transcription was performed at 37 °C for 16 h or at 42 °C for 3 h in a reaction mixture containing 40 mM Tris-HCl (pH 8.0), 10 mM dithiothreitol, 2 mM spermidine, 8 mM MgCl_2_, 2.5 mM each NTP, template DNA (~0.2 mg/mL), and pure T7 RNA polymerase (~100 µg/mL) [20,21]. The transcripts were purified by denaturing 12% polyacrylamide gel electrophoresis. The concentrations of the obtained RNAs were determined from the ultraviolet absorbance at a wavelength of 260 nm using NanoPhotometer (Implen, München, Germany).

### 2.2. Expression and Purification of E. coli AlaRS442N

The plasmid (pET20b) coding *Escherichia coli* AlaRS442N (N-terminal fragment with 442 residues of *E. coli* AlaRS) is a gift from Dr. Paul Schimmel (The Scripps Research Institute). *E. coli* BL21-Codon Plus (DE3)-RIL (Stratagene, La Jolla, CA, USA) containing the *E. coli* AlaRS442N was grown and induced with 0.5 mM isopropyl-D-thiogalactoside (IPTG). Cultures were harvested and cells were suspended in lysis buffer (20 mM Tris-HCl (pH 8.0), 500 mM NaCl, 10 mM imidazole), followed by the addition of lysozyme and Triton X-100, and then the cells were disrupted by sonication on ice. The supernatant was charged onto a Ni-NTA agarose (QIAGEN, Valencia, CA, USA) column equilibrated with lysis buffer and washed with wash buffer (20 mM Tris-HCl (pH 8.0), 500 mM NaCl, and 20 mM imidazole), and His-tagged AlaRS442N was eluted with elution buffer (20 mM Tris-HCl (pH 8.0), 500 mM NaCl, and 250 mM imidazole). Fractions containing the protein were pooled and dialyzed twice in 1 L of dialysis buffer (40 mM Tris-HCl (pH 8.0), 200 mM NaCl, 0.2 mM EDTA) followed by concentration by Amicon Ultra Ultracel-30K (Merck Millipore, Billerica, MA, USA). Finally, the enzymes were stored in 50% glycerol.

### 2.3. Electrophoretic Mobility Shift Assay

9 µL of RNA solution in ACCA reaction buffer (50 mM HEPES-KOH (pH 7.5), 400 mM KCl, 20 mM Mg(OAc)_2_) was prepared by heating 3 µL of 500 µM of each RNA in water at 95 °C for 10 min, placing on ice for 10 min, and then combining, followed by the addition of 3 µL of 3 × ACCA reaction buffer (and 3 µL of water if necessary). After being incubated on ice for 2 h and the addition of 1 µL of 10 × loading buffer (150 mM Mg(OAc)_2_, 50% glycerol), the solution (final 150 µM) was analyzed by electrophoresis at 4 °C through nondenaturing 8% polyacrylamide gels in THM running buffer (50 mM Tris-HCl (pH 7.5), 100 mM KCl, 10 mM Mg(OAc)_2_). The gel was stained with 0.04% toluidine blue [21,22].

### 2.4. Preparation of Alanyl-Minihelix^Ala^

The aminoacylation reaction was performed at 37 °C in a reaction mixture containing 50 mM HEPES-NaOH (pH 7.4), 10 mM MgCl_2_, 30 mM KCl, 2 mM dithiothreitol, 2 mM ATP, and 10 µM L-alanine, with 5 µM RNA minihelix^Ala^, and 2 mg/mL *E. coli* AlaRS442N. After incubation for 8 min, the alanylated minihelix^Ala^ was prepared according to Sardesai et al. [23]. The aminoacylation reaction was also confirmed by using L-[U-^14^C]alanine (132.0 mCi/mmol) (Moravek, Inc., Brea, CA, USA) instead of non-radiolabeled L-alanine, by spotting the aliquots onto trichloroacetic acid-soaked filter pads, washing with cold 5% trichloroacetic acid, and measuring by scintillation counting [24].

### 2.5. Peptidyl Transfer Reaction Using Alanyl-Minihelix^Ala^

1000 pmol of P1c2 or P1c2^UGGU^ was dissolved in 2 µL of ACCA reaction buffer. After heating at 95 °C for 10 min, it was incubated on ice for 60 min to be self-dimerized. Then, 1000 pmol of alanyl-minihelix^Ala^ was added and placed at 4 °C for 24 h. The reaction product was treated with 0.22 µL of 250 mM KOH (pH of the solution was changed from 7.5 to pH 9.0) at room temperature for 45 min to liberate any alanylalanine that might form. As a negative control, 2 µL of ACCA reaction buffer was used instead of 2 µL of 1000 pmol of P1c2 or P1c2^UGGU^ in the first step.

### 2.6. Mass Spectrometry

Each sample in the previous section was diluted by water up to 10 µL. The final product, alanylalanine, was characterized by LCMS-2010EV electrospray mass spectrometry (Shimadzu, Kyoto, Japan) in positive-single ion monitoring (SIM) mode at *m*/*z* 199.20 using the analytical column Luna C8(2) 100 Å (150 mm × 4.6 mm, Phenomenex). The mobile phase consisted of 65% methanol. The flow rate was set at 0.2 mL/min and the injection volume was 8 µL. The column temperature was 60 °C. The peak area was calculated according to the protocol of the manufacturer (Shimadzu, Kyoto, Japan).

## 3. Results

### 3.1. Gel Shift Assay of P1c2, P1c2^UGGU^, P1c2^UUUU^, and Minihelix^Ala^

**Figure 3:**
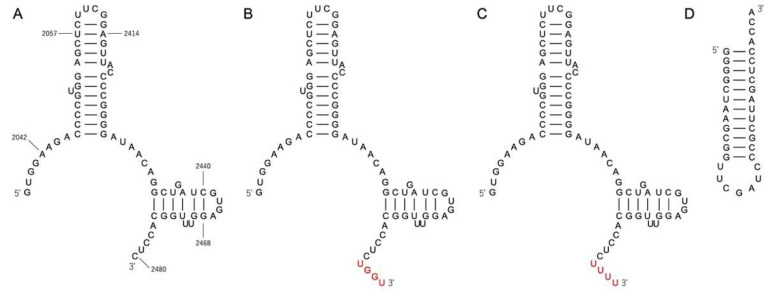
The sequences and the secondary structures of (**A**) P1c2, (**B**) P1c2^UGGU^, (**C**) P1c2^UUUU^, and (**D**) minihelix^Ala^. The secondary structures of (**A**–**C**) are derived from the region of the C-loop. The numbering in (**A**) is from *Deinococcus radiodurans* 23S rRNA, and P1c2 is obtained from the modification of P1c construct [15].

P1c, a mutant of P1, is derived from the large ribosomal subunit of *D. radiodurans* and has been reported to exhibit higher self-dimerization ability [15]. In this study, we prepared modified P1c constructs, P1c2 (70-mer), P1c2^UGGU^ (74-mer), and P1c2^UUUU^ (74-mer) (Figure 3). P1c2^UGGU^ and P1c2^UUUU^ possess additional UGGU and UUUU at the 3′-end of P1c2, respectively (Figure 3B,C). The UGGU terminal segment of the P1c2^UGGU^ was an experimental addition to the original P1c2, specifically designed for the purpose of tethering the terminal ACCA of the minihelices (Figure 3B). Minihelix^Ala^ (35-mer) is derived from *E. coli* tRNA^Ala^ (Figure 3D) [25,26].

**Figure 4:**
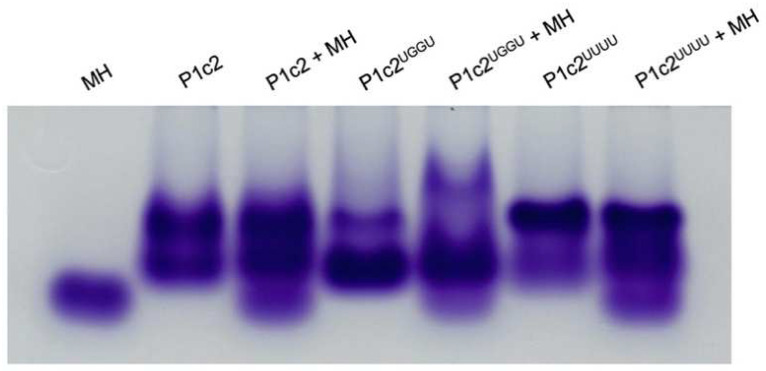
Electrophoretic mobility shift assay of P1c2, P1c2^UGGU^, and P1c2^UUUU^ in the presence and absence of minihelix^Ala^ (MH) in THM running buffer.

P1c2, P1c2^UGGU^, and P1c2^UUUU^ all showed dual mobility shifts in native gel electrophoresis (Figure 4). The result indicates the partial self-dimerization of each molecule under the experimental conditions. However, P1c2^UGGU^ with minihelix^Ala^ showed a different mobility shift from that of P1c2 with minihelix^Ala^, P1c2^UUUU^ with minihelix^Ala^, and P1c2^UGGU^ alone, suggesting the binding of minihelix^Ala^ to dimeric P1c2^UGGU^ (Figure 4).

### 3.2. Attempt and Detection of Peptide Bond Formation

**Figure 5:**
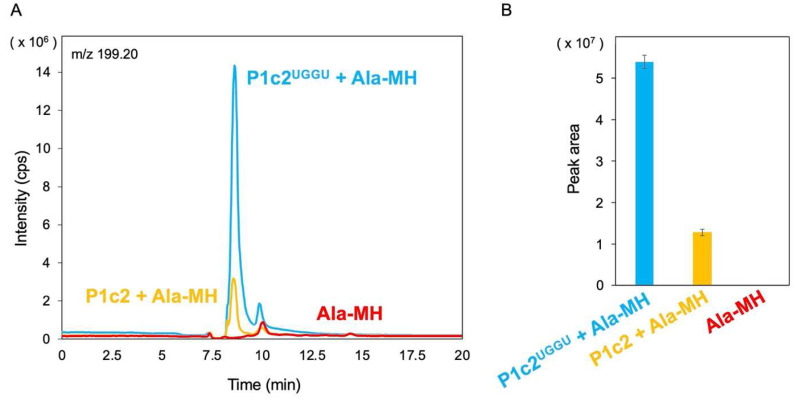
(**A**) LC/MS electrospray mass spectrometry of reaction products from P1c2^UGGU^ and alanyl-minihelix^Ala^ (Ala-MH) (light blue), P1c2 and Ala-MH (bright yellow), and Ala-MH alone (red). The spectra were recorded in positive-single ion monitoring (SIM) mode at m/z 199.20. The peak corresponds to the potassium ion adduct of alanylalanine. (**B**) Peak area showing LCMS-2010EV electrospray mass spectrometry intensities in reaction products from P1c2^UGGU^ and Ala-MH (light blue), P1c2 and Ala-MH (bright yellow), and Ala-MH alone (red). Error bars represent the standard deviation of triplicate experiments.

*E. coli* AlaRS442N is known to be responsible for the synthesis of alanyl-minihelix^Ala^ as well as alanyl-tRNA^Ala^ [27]. Using the AlaRS442N, alanyl-minihelix^Ala^ was prepared as described above. Then, P1c2^UGGU^ was incubated with alanyl-minihelix^Ala^ in an attempt to form a peptide bond between the α-amino nitrogen of alanine of one alanyl-minihelix^Ala^ and the carbonyl carbon of alanine, which is esterified to the 3′-terminal adenosine of another minihelix. We incubated alanyl-minihelix^Ala^ with P1c2^UGGU^, or with P1c2 (Figure 5A), and analyzed any alanylalanine that might form by liberation from minihelix^Ala^ by alkali treatment. As a negative control, alanyl-minihelix^Ala^ was also incubated without further addition of RNA (Figure 5A). The peaks of the MS chromatogram at *m*/*z* 199.20 (corresponds to the potassium ion adduct of alanylalanine) were detected in the case of alanyl-minihelix^Ala^ with P1c2^UGGU^, alanyl-minihelix^Ala^ with P1c2, or alanyl-minihelix^Ala^ alone (Figure 5A). The peak height was much larger in the case of alanyl-minihelix^Ala^ with P1c2^UGGU^ than in the case of alanyl-minihelix^Ala^ with P1c2 (Figure 5A). About a 4.2-fold increase in the peak area was obtained in alanyl-minihelix^Ala^ with P1c2^UGGU^ compared to alanyl-minihelix^Ala^ with P1c2 (Figure 5B). In contrast, an apparent peak was not detected in the case of alanyl-minihelix^Ala^ alone (Figure 5).

## 4. Discussion

The crystal structure of the large ribosomal subunit of *D. radiodurans* showed that the PTC is formed by a pocket-like symmetrical RNA dimer that is composed of two L-shaped RNA units (Figure 2A,B) [3,11,12,13,14]. The symmetrical region is highly conserved, both structurally and phylogenetically, across kingdoms [28]. The L-shaped RNA is similar in size to tRNA (Figure 2D). Yonath’s group recently showed that the conserved pocket region could catalyze the peptide bond formation with CCA-pcb as a P-site substrate and C-Pmn as an A-site substrate [16].

Minihelix-like hairpin RNAs are the most fundamental short RNAs in evolution [3,29,30,31,32,33,34,35]. In addition, a hairpin RNA with NCCA-3′ may also be related to the origin of homochiral aminoacylation in the RNA world [3,36,37,38,39]. Kissing-loop interaction-mediated conformational changes may have contributed to the formation of the minihelix-like structure, and tRNA could have originated from further kissing-loop interactions between two minihelix-like RNA molecules [22]. These results suggest that not only tRNA, but also rRNA forming the PTC might be derived from a half-tRNA-like minihelix. G3:U70 base pair in the RNA minihelix might be a vestige of the primordial genetic code, which may be called “operational RNA code” [3,29,30]. It has been shown that the α-subunit of alanyl-tRNA synthetase (AlaRS-α) of *Nanoacrchaeum equitans* performs G3:U70-independent alanylation of the RNA minihelix, indicating the existence of a simplified phase in AlaRS evolution [39].

**Figure 6:**
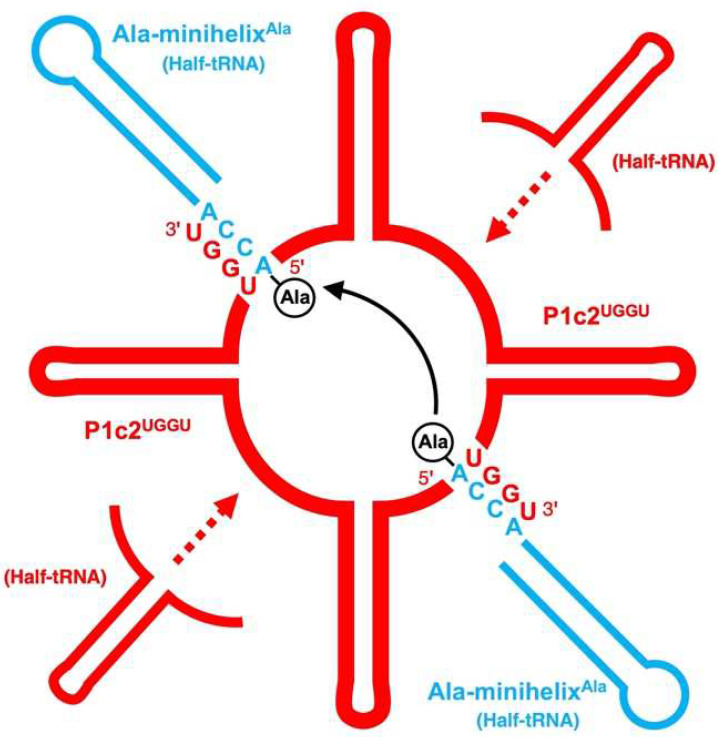
Schematic and conceptual representation of peptide bond formation between two alanyl-minihelices^Ala^, using the dimerized P1c2^UGGU^ as a scaffold. Appended UGGU sequence of P1c2^UGGU^ makes the interaction with ACCA of minihelix^Ala^ possible, bringing two alanine residues in close proximity. Some of half-tRNAs would have evolved to the PTC, and others (minihelices) to tRNAs.

On the ribosome, the “two” CCA termini of tRNAs must be positioned in close proximity [9], resembling the positioning of the index fingers in “Goromaru pose” [10]. Considering these things comprehensively, a scaffold for the necessary proximity of two aminoacyl-tRNAs in the course of biological/evolutionary continuity should be identified. Here, we have shown that a dipeptide (alanylalanine) is generated between two alanyl-minihelices^Ala^ using P1c2^UGGU^ as a scaffold (Figure 5). P1c2^UGGU^ forms a dimer and minihelix^Ala^ interacts with it. However, the ratio of the dimer to the monomer was lower for P1c2^UGGU^ than for the P1c2 and P1c2^UUUU^ (Figure 4). It is also likely that the monomer also binds to the minihelix. Therefore, it seems possible that there are two different dimer forms, one is active and the other is inactive, which show an equal gel mobility, and that the UGGU end suppresses formation of the inactive dimer (Figure 4). In any case, the effect of P1c2^UGGU^ on peptide bond formation was clear, because the additional UGGU could interact with ACCA of minihelix^Ala^, causing the two alanine residues to be in close proximity, as schematically shown below (Figure 6). While we cannot concretely specify the positioning of the two alanine residues of minihelices^Ala^ in the presence of self-dimerized P1c2^UGGU^ because of lack of tertiary structures of these complexes, the complementary interaction between UGGU of P1c2^UGGU^ and ACCA of alanyl-minihelices^Ala^ was apparently effective for peptide bond formation (Figure 5).

In the crystal structure of the symmetrical region within the C-loop of domain V of *D. radiodurans* 23S rRNA (PDB ID: 1NJP) [40], the distance between the 3′-ends of P-core unit and A-core unit (labeled with circled 3′ in Figure 2B) is ~20 Å. In DNA-templated chemical synthesis, Gartner and Liu investigated whether the reaction rate correlates with the number of residues in a single chain in DNA-promoted conjugated addition reactions and nucleophilic substitution reactions. They showed that the rate of this bimolecular reaction did not change remarkably even if the substrates were separated by a flexible linker with an approximate length of 30 residues [41]. The ring-closure probability of DNA fragments having cohesive ends has also been studied, and the peak of the probability is at approximately 15 nucleotides. Even at 30 nucleotides, the probability remains approximately half of the peak value [42]. These results also suggest that even if two alanine residues are separated in the PTC model complex used in our experiments, the reaction could occur as long as the complementary interaction between UGGU of P1c2^UGGU^ and ACCA of alanyl-minihelices^Ala^ tether them within a permitted distance.

Peptide bond formation using alanyl-minihelix^Ala^ with P1c2 was markedly reduced compared with that of alanyl-minihelix^Ala^ with P1c2^UGGU^ (Figure 5). This result could be due to the lack of interaction between UGGU and ACCA in the prior case. However, the ACCA of alanyl-minihelix^Ala^ could interact with the self-dimerized P1c2 in a nonspecific manner. Alternatively, part of specific interactions derived from the CCA and the PTC may be reflected because CCA-pcb and C-Pmn as the P and A-site analogs, respectively, can be substrates in the similar reaction [16]. These analogs are chemically activated artificial molecules that are different from aminoacylated RNAs. In contrast to the case with P1c2^UGGU^, alanyl-minihelix^Ala^ alone, without any scaffold RNAs, did not show an apparent formation of alanylalanine (Figure 5). These results indicate the importance of the scaffold in this reaction. An artificial model system with puromycin attached to UGGU through a 5ʹ-5ʹ phosphodiester linkage also resulted in peptide bond formation from aminoacyl-minihelices using a UGGU-ACCA interaction [43]. In both cases, the underlying chemistry is the thermodynamically downhill reaction that occurs from the conversion of high-energy aminoacyl (ester) bonds to low-energy peptide (amino) bonds by satisfying the proximity of two aminoacyl-esters.

The CCA sequence of tRNA is important for the peptidyl transfer reaction within the ribosome [3,8]. The specific Watson–Crick base-pairing of C74 and C75 of peptidyl-tRNA with G2252 and G2251 in the P-loop of 23S rRNA (*E. coli* numbering), respectively, and that of C75 of aminoacyl-tRNA with G2553 in the A-loop of 23S rRNA (*E. coli* numbering) is essential for its activity [5]. P1c2^UGGU^ lacks these regions (Figure 3B) but restraining two aminoacylated RNAs into fairly close contact in an RNA environment may be sufficient to form a peptide bond. Functional interaction sites might have been revised in the process of RNA evolution. In fact, many functional insertions were identified in *E. coli* rRNAs, revealing the architecture of *E. coli* rRNAs to be substantially flexible and tolerant [44]. The interactions seen in the current ribosome render the tethering effect of two tRNAs (CCAs) on the PTC, and are “conceptually” similar to the effect found in this study, although the parts that interact with CCA of tRNAs on the ribosome are not necessarily located on contiguous sequences of 23S rRNA.

It is not clear whether the structure/sequence of P1c2^UGGU^ is essential for stimulation above the basal proximity rate or not, because there is no experimental control to indicate PTC function. However, from a continuity perspective in biological evolution, it is worthy of note that the two symmetrically arranged tRNA-like units seen in the large ribosomal subunit actually function by scaffolding aminoacyl-minihelices for peptide bond formation. General purpose as a “high-speed protein synthesis machine” of the ribosome might not have been necessary in early contexts of evolution of the protein synthesis system. The rationale for starting simple might indicate that the early minihelices would be able to dimerize by themselves through some kind of complementary stretches. The first functional RNA comprised of tRNA-like molecules may have been formed by the duplication of small RNAs. With the same origin, some of the molecules might have evolved to contribute to the formation of PTC. The ribosome may be largely entropic in origin and may have started mainly to position the substrates in proximity during peptidyl transfer [45]. Then, the PTC could have further evolved into the modern ribosome by adding more RNA portions and surrounding proteins, enabling water exclusion within the active site and the “proton shuttle” for effective peptide bond formation [11,46,47]. Other molecules may have evolved to function as real tRNAs (Figure 2D and Figure 6). Aminoacyl-tRNA synthetases (aaRSs) and ribosomal subunits (large and small) are functionally separate. The minihelix is a half-sized tRNA molecule and this region of tRNA is primitive and interacts with the conserved domain of aaRSs and the large ribosomal subunit. In contrast, the other tRNA half (including the anticodon region), that interacts with the non-conserved domain of aaRSs and the small ribosomal subunit, would have been added later [3]. In that sense, our experiments do not assume the interaction of alanyl-minihelix^Ala^ and a mRNA template. Thus, primordial tRNA-like molecules (not real tRNAs) of the same origin may have evolved (Figure 6). Based on the knowledge of the tRNA-rRNA homologies [48], tRNA dimers may also have been mimics of the ribosome [49]. On the other hand, faced with the metabolic paradoxes of purine and pyrimidine biosynthesis, i.e., purine and pyrimidine rings are synthesized from glycine and aspartic acid, respectively [50], one possibility may be peptide nucleic acids (PNA) as a precursor to RNA [51].
